# The impact of universal National Health Insurance on population health: the experience of Taiwan

**DOI:** 10.1186/1472-6963-10-225

**Published:** 2010-08-04

**Authors:** Yue-Chune Lee, Yu-Tung Huang, Yi-Wen Tsai, Shiuh-Ming Huang, Ken N Kuo, Martin McKee, Ellen Nolte

**Affiliations:** 1 Institute of Health and Welfare Policy, College of Medicine, National Yang-Ming University, Taipei, Taiwan; 2 Department of Gerontological Care and Management, Chang Gung Institute of Technology, Tao-Yuan, Taiwan; 3 Division of Health Policy Research and Development, Institute of Population Health Sciences, National Health Research Institutes, 35 Keyan Road, Zhunan, Miaoli County 35053, Taiwan; 4 Office of Statistics, Department of Health, Taipei, Taiwan; 5 European Centre on Health of Societies in Transition, London School of Hygiene and Tropical Medicine, London, UK; 6 Director Health and Healthcare, RAND Europe, Cambridge, UK

## Abstract

**Background:**

Taiwan established a system of universal National Health Insurance (NHI) in March, 1995. Today, the NHI covers more than 98% of Taiwan's population and enrollees enjoy almost free access to healthcare with small co-payment by most clinics and hospitals. Yet while this expansion of coverage will almost inevitably have improved access to health care, however, it cannot be assumed that it will necessarily have improved the health of the population. The aim of this study was to determine whether the introduction of National Health Insurance (NHI) in Taiwan in 1995 was associated with a change in deaths from causes amenable to health care.

**Methods:**

Identification of discontinuities in trends in mortality considered amenable to health care and all other conditions (non-amenable mortality) using joinpoint regression analysis from 1981 to 2005.

**Results:**

Deaths from amenable causes declined between 1981 and 1993 but slowed between 1993 and 1996. Once NHI was implemented, the decline accelerated significantly, falling at 5.83% per year between 1996 and 1999. In contrast, there was little change in non-amenable causes (0.64% per year between 1981 and 1999). The effect of NHI was highest among the young and old, and lowest among those of working age, consistent with changes in the pattern of coverage. NHI was associated with substantial reductions in deaths from circulatory disorders and, for men, infections, whilst an earlier upward trend in female cancer deaths was reversed.

**Conclusions:**

NHI was associated in a reduction in deaths considered amenable to health care; particularly among those age groups least likely to have been insured previously.

## Background

Taiwan established a system of universal National Health Insurance (NHI) in March, 1995, replacing 13 occupational funds that had covered only about 60% of the population (including military health services), largely covering the population at working age. Coverage was 14%, 77%, and 57% for those aged under 20, 20-64 and 65 year and over respectively. Today, the NHI covers more than 98% of Taiwan's 23 million population and enrollees enjoy almost free access to healthcare, except for a small co-payment, plus some registration fees that, although not mandatory, are required by most clinics and hospitals. Yet while this expansion of coverage will almost inevitably have improved access to health care, given evidence of significant disparities in utilization under the former system [1], it cannot be assumed that it will necessarily have improved the health of the population. Indeed, Levy and Meltzer [2] and McWilliams [3] have drawn attention to the many existing limitations of existing research linking expansion of coverage to health improvement. In this paper we take advantage of the opportunity provided by the natural experiment of implementing NHI in Taiwan to examine this issue.

Taiwan's NHI scheme has been described previously [4,5]. In brief, it is a universal single-payer social health insurance system, based on a public-contract model, offering comprehensive benefits. The main sources of funds are an income-related premium (payroll taxes), with employees, employers, and government all paying a share of premiums. The share of the premiums paid by the insured, by employers, and by government varies according to employment status. For employees in public or private enterprises, the shares are 30%, 60% and 10% respectively. The high-income self-employed insured pay 100% of the premium themselves. The government pays 100% of the premium for military personnel and the low-income unemployed, and pays 40% of the premium for the other unemployed. The amount collected by the NHI in respect of each enrollee varies according to his or her number of dependents, although dependents in excess of three are effectively insured for free. These insurance premiums are supplemented by smaller contributions from co-payments, alcohol and tobacco taxes and lottery revenues, and recompense from national environmental pollution funds and automobile insurance. In 2008, total national health expenditure was US$ 23.9 billion, or 6.4% of GDP (exchange rate 33 NTD:1USD). Expenditure on the NHI was US$12.8 billion, or 53.5% of national health expenditure, which equated to 3.4% of gross domestic product (GDP) [6], which was lower than in most OECD countries [7].

Previous evaluations of Taiwan's NHI have focused on access and cost containment [8-10], with little research on the impact of its introduction on population health. One study found that the NHI had reduced rural-urban disparities in the incidence of ruptured appendicitis [11]. Another found improved access to care by the elderly but no measurable impact on their self-perceived health [12]. Yet another examined changing life expectancy [13], but it failed to identify an overall association with the introduction of NHI although the gap between the most and least healthy townships did narrow significantly. However, as life expectancy captures the entire range of health determinants [14], more specific analyses are needed.

In this paper we seek to identify any contribution of health care using the concept of amenable mortality, or "mortality amenable to health care", applying methods developed by Nolte and McKee [15]. These capture causes of deaths that should not occur in presence of timely and effective health care [16] and have been used in other evaluations of health system performance [17-19].

We hypothesized that, if the introduction of NHI had an impact on population health, this should be reflected by a discontinuity in the trend in amenable mortality but not in non-amenable mortality. We also hypothesized that the effect of NHI would be less apparent among the working age population, who were more likely to have been insured previously than non-working age groups. With the advantage of comprehensive high quality death registration data covering a population of almost 23 million, our findings may be relevant to other countries that have yet to establish universal health insurance coverage.

## Methods

We used joinpoint regression analysis [20] to identify changes in mortality trends that might be associated with implementation of NHI in Taiwan, distinguishing mortality from causes considered amenable to health care and mortality from all other causes during 1981-2005.

### Data

Mortality data were taken from the Death Registry Database, maintained by the Taiwan Department of Health, with population data from the Household Population Registration System (HPRS) provided by the Ministry of the Interior. These were used to calculate age, sex and cause-specific mortality rates, coded using the ninth revision of the *International Classification of Diseases and Deaths *(ICD-9).

### Amenable mortality

Causes of death considered amenable to health care are those defined by Nolte and McKee [15,17]. They include causes such as bacterial infections, treatable cancer, diabetes, cardiovascular and cerebrovascular disease, and complications of common surgical procedures for people under age 75.

We combined causes of deaths to calculate cause-specific amenable mortality rates. We also include ischemic heart disease (IHD) but treat this cause separately as the precise contribution of health care to reductions in deaths from this condition remains unresolved [21,22]. Also, deaths from IHD tend to dominate analyses because of their large numbers. For consistency with other studies, we here present amenable deaths with and without 50% IHD [17,19].

The number of deaths from causes other than amenable at ages 0-74 was 40,352 in 1981, rising to 55,141 in 2005 while the corresponding figures for amenable causes (excluding IHD) fell, from 21,508 to 16,441, with deaths from IHD rising from 1,643 to 3,338 in the same period.

### Measurement and analysis

**Figure 1:**
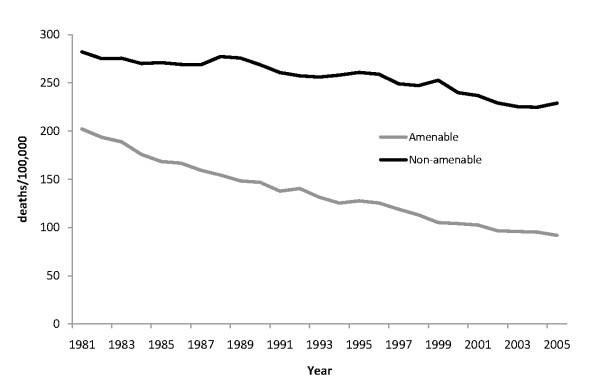
**Trends in amenable and non-amenable mortality age 0-74 in Taiwan, 1981-2005**.

To account for changes in population composition, we calculated age-standardized mortality rates (ASMR) using direct standardization to the 2000 WHO world standard population. The ASMR is the sum of the product of mortality rate in the ith age stratum (*M_i_*) in a given year in Taiwan and the proportion of population of standard (WHO) population in the same age stratum in the same year *(StdP_i_) *as follows: $ASMR=\overset{n}{\underset{i=1}{\sum }}[M_{i}\times StdP_{i}]$ Analyses were undertaken for all ages 0-74 as well as for three age groups, under 20, 20-64, and 65 years and over. Trends in amenable and non-amenable mortality are shown in Figure 1.

### Statistical analyses

We used joinpoint regression analysis originally developed for use in cancer epidemiology, to identify points of significant inflection in trends. The analysis starts with minimum number of inflections (joinpoints), and tests whether one or more additional joinpoints should be added to the model. In the final model, each joinpoint indicates a statistically significant change, either increase or decrease, in trend [23]. We selected the Bayesian information criterion approach, which identifies the model with the best fit by penalizing the cost of extra parameters. The annual percentage change (APC) is calculated for the time segments on either side of inflection points. Joinpoint regression analyses were performed using Joinpoint version 3.2 software obtained from the US National Cancer Institute [23].

## Results

### Trends in total amenable mortality

Table 1 shows the results of the joinpoint analysis. For all causes of deaths, the decline in the mortality rate was significant before implementation of the NHI. It became briefly insignificant (in both sexes combined and males alone) and again significant immediately after implementation of NHI, at a similar rate of decline to before NHI.

**Table 1:** Joinpoint regression analyses on age-standardized amenable mortality rates in Taiwan, 1981-2005

	Trend 1		Trend 2		Trend 3		Trend 4		Trend 5	
	
Mortality	Time Period	APC	Time Period	APC	Time Period	APC	Time Period	APC	Time Period	APC
All causes										
Both genders	1981-1993	-1.74*	1993-1996	-0.57	1996-2003	-2.50*	2003-2005	0.06		
Females	1981-1996	-2.22*	1996-2005	-2.72*						
Males	1981-1984	-2.36*	1984-1993	-1.23*	1993-1996	-0.09	1996-2003	-2.25*	2003-2005	1.08

Amenable causes										
Both genders	1981-1993	-4.38*	1993-1996	-0.53	1996-1999	-5.83*	1999-2005	-2.77*		
Females	1981-1993	-4.45*	1993-1996	-1.48	1996-1999	-5.70*	1999-2005	-3.21*		
Males	1981-1987	-5.05*	1987-1993	-3.61*	1993-1996	-0.53	1996-1999	-5.77*	1999-2005	-2.45*

Other causes (non-amenable causes)										
Both genders	1981-1999	-0.64*	1999-2003	-2.74*	2003-2005	1.04				
Females	1981-1989	-0.48	1989-2005	-1.80*						
Males	1981-1999	-0.37*	1999-2003	-2.47*	2003-2005	1.72				

Amenable causes with 50% IHD										
Both genders	1981-1993	-4.04*	1993-1996	-0.81	1996-1999	-5.67*	1999-2005	-2.61*		
Females	1981-2005	-3.86*								
Males	1981-1987	-4.66*	1987-1993	-3.18*	1993-1996	-0.78	1996-1999	-5.50	1999-2005	-2.22*

IHD										
Both genders	1981-1992	1.78*	1992-2005	-2.61*						
Females	1981-1992	1.51	1992-1998	-6.73	1998-2005	-2.66				
Males	1981-1992	2.29*	1992-2005	-1.63*						

*Note*. APC= annual percent change; IHD= ischemic heart disease.

* APC is statistically significantly different from zero (2-sided at *p *< .05).

Turning to amenable mortality for men and women combined, the joinpoint analysis identified three significant inflection points, generating four distinct trends between 1981 and 2005. A steady decline from 1981 (APC = -4.38, p < .05) was arrested in 1993 (APC = -0.53, p = .84) but then accelerated downward after 1996 (APC = -5.83, p < .05) before slowing once more in 1999 (APC = -2.77, p < .05). When analyzed separately, a similar trend was identified for women while for men an additional inflection point was identified in 1987 (APC = -3.61, p < .05). Mortality from other causes fell consistently for both sexes between 1981 and 1999 (APC = -0.64, p < .05), although at a somewhat slower rate compared to amenable mortality; it accelerated downward after 1999 (APC = -2.74, p < .05), arrested in 2003 and thereafter (APC = 1.04, p = .65). Similar trends were found in men but for women only one inflection point was identified in 1989, following a smaller decline in the previous period.

Table 1 further shows amenable mortality with 50% of deaths from IHD included. For men and women combined (and also for men alone), the pattern was similar to that seen for amenable mortality without 50% IHD; but for females, no inflection point was identified. Further analysis using IHD deaths alone for men and women combined reveals that a decelerating increase between 1981 and 1992 (APC = -1.78, p < 0.05) was reversed in 1992 (APC = -2.61, p < .05). Similar trends were found in men but for women, an additional inflection point was identified in 1998 (APC = -2.66, p > .05), following an accelerated downward trend after 1992 (APC = -6.73, p < .05).

### Trends of amenable mortality by age

Tables 2 stratifies amenable mortality by age groups for both sexes. It suggests that the effect of NHI implementation was more apparent among those under age 20. A declining trend in amenable mortality briefly reversed between 1992 or 1993 and 1996, followed by a significant decrease thereafter (APC = -5.84). For those at working age (20-64 years) there was no significant effect of NHI except for the period 1999-2005. A similar pattern was seen for those aged 65 and over, although the magnitude of the decline in amenable mortality during 1999-2005 (APC = -2.91, p < .05) was higher than among those of working age (APC = -1.21, p < .05).

**Table 2:** Joinpoint regression analyses on amenable mortality rates by age-group in Taiwan, 1981-2005

	Trend 1		Trend 2		Trend 3		Trend 4		Trend 5	
	
Age group	Time Period	APC	Time Period	APC	Time Period	APC	Time Period	APC	Time Period	APC
Under 20 years	1981-1987	-11.64*	1987-1993	-3.29*	1993-1996	12.36	1996-2005	-5.84*		
20-64 years	1981-1987	-4.63*	1987-1996	-2.27*			1996-1999	-4.75	1999-2005	-1.21*
65 years and over	1981-1989	-4.06*	1989-1993	-6.33*	1993-1996	-1.31	1996-1999	-5.29	1999-2005	-2.91*

*Note*. APC= annual percent change.

* APC is statistically significantly different from zero (2-sided at *p *< .05).

### Trends of amenable mortality by sub-group amenable causes

Table 3 summarizes the analyses for major amenable causes of death. For circulatory diseases, the picture was similar to that for all amenable causes. On average, the ASMR declined significantly (10.19%, 8.63% and 9.13% per year) among women, men, and both combined respectively, following the introduction of NHI (1996-1999). These trends continued at a slower, but still significant rate during 1999-2005. The ASMR for treatable cancers increased significantly during 1990-97. Soon after the introduction of NHI, the trends for women (APC = -1.49, p < .05) and for both sexes together (APC = -0.75, p < .05) began to reverse, significantly so, during 1997-2005. The trend for men continued to increase, although at a slower rate, becoming insignificant during 1997-2005. Trends in infectious diseases showed no clear pattern except for a significant decline after 1997 for men (APC = -10.03, p < .05). There was no significant change in trends of respiratory disease and genitourinary disease mortality that can be associated with the introduction of NHI.

**Table 3:** Joinpoint regression analyses on sub-group age-standardized amenable mortality rates in Taiwan, 1981-2005

	Trend 1		Trend 2		Trend 3		Trend 4		Trend 5	
	
Diseases	Time Period	APC	Time Period	APC	Time Period	APC	Time Period	APC	Time Period	APC
Circulatory^a^	1981-1984	-3.25*	1984-1993	-6.35*	1993-1996	-3.80	1996-1999	-9.13*	1999-2005	-4.12*
Females	1981-1988	-5.11*	1988-1993	-8.53*	1993-1996	-5.40	1996-1999	-10.19*	1999-2005	-5.51*
Males	1981-1983	-1.13	1983-1993	-5.75*	1993-1996	-1.91	1996-1999	-8.63*	1999-2005	-3.33*
Infections^b^	1981-1986	-9.05*	1986-1989	2.62	1989-1993	-5.47*	1993-2005	-9.65*		
Females	1981-1985	-8.78*	1985-1992	-0.99	1992-2005	-10.82*				
Males	1981-1986	-9.37*	1986-1989	2.95	1989-1997	-7.10*	1997-2005	-10.03*		
Cancer^c^	1981-1991	0.29	1991-1997	2.96*	1997-2005	-0.75*				
Females	1981-1990	0.63*	1990-1997	2.11*	1997-2005	-1.49*				
Males	1981-1991	-0.73	1991-1997	4.56*	1997-2005	0.77				
Respiratory^d^	1981-1984	-16.06*	1984-1990	4.43	1990-1993	-12.52	1993-2005	-0.09		
Females	1981-1984	-17.45*	1984-1990	2.34	1990-1994	-12.80	1994-2005	-1.04		
Males	1981-1983	-22.29	1983-1990	4.13	1990-1993	-10.16	1993-2005	0.53		
Genitourinary^e^	1981-1989	-5.61*	1989-1995	1.96	1995-1999	-4.75	1999-2002	3.36	2002-2005	-2.83
Females	1981-1988	-5.78*	1988-2005	-1.13*						
Males	1981-1989	-5.47*	1989-1995	3.13*	1995-1999	-4.23	1999-2005	0.84		

*Note*. APC= annual percent change.

^a ^include chronic rheumatic heart disease, hypertensive disease, and cerebrovascular disease

^b ^include intestinal infections, tuberculosis, other infections (diphtheria, tetanus, poliomyelitis), whooping cough, septicemia, and measles

^c ^include colon and rectum, skin, breast, cervix uteri, cervix uteri and body of uterus, testis, Hodgkin's disease, and leukemia

^d ^include all respiratory diseases, influenza, and pneumonia

^e ^include nephritis and nephrosis, and benign prostatic hyperplasia

* APC is statistically significantly different from zero (2-sided at *p *< .05).

## Discussion

In this study we assessed changes in amenable mortality before and after implementation of universal health insurance coverage in Taiwan. We find that the introduction of NHI was associated with a significant acceleration in the rate of decline of causes of death considered amenable to health care. In contrast, there was no clear change in the trend of mortality from conditions not considered amenable to health care that could be associated with the introduction of NHI. These findings are in general consistent with our hypothesis and with studies reviewed by Levy and Meltzer which, while noting methodological limitations, found that improved health insurance coverage was associated with improved health [2,3,24-27].

Our study also found that the association with the introduction of NHI was more apparent among the young, aged less than 20 years, followed by the elderly, and least among the working age population. This result is consistent with our expectations as 77% of the working age population were already covered by the pre-existing social insurance; thus they were inevitably going to be affected less by the introduction of NHI. In contrast, most children, except children of government employees, were previously uninsured; only 14% of those aged 20 had social insurance coverage and therefore had more to gain.

Our analysis did not find substantial benefits accruing to elderly people but it must be recalled that amenable deaths occur, by definition, below the age of 75 years. This is for a number of reasons, including the problems of coding often multiple co-existing causes contributing to death, but it does constrain the power of our study to identify a true effect. Furthermore, unlike with children, where coverage was very low, 57% of elderly people were already covered by social insurance before the introduction of NHI. In addition, elderly people in poor health may have selectively enrolled in either the Indigent Health Insurance or Farmers Health Insurance (FHI) schemes introduced in 1989, or fraudulently used vouchers of people with insurance coverage to visit a doctor, something that was not uncommon in the previous system. Taken together, these factors may explain the weak association between introduction of NHI and health gain among the elderly. However, our finding that the effect attributed to NHI varied among people with different levels of social insurance coverage supports the view that the decline in amenable mortality was related to introduction of NHI.

Our finding of a significant effect of NHI on population health may seem to contradict previous studies in Taiwan that were unable to demonstrate a significant gain in life expectancy a decade after the introduction of NHI [13] or significant changes in perceived health status among the elderly [12]. However, the present study uses a more specific measure of the contribution of health care [15,17] whereas changes in life expectancy and perceived health status are likely to reflect a broader spectrum of health determinants including changes in life style, environmental and biological factors. Furthermore, the application of joinpoint regression in this study allows us to identify inflection points and relate them to the introduction of NHI, something not possible with the earlier studies that simply compared before and after findings.

Our study is also inconsistent with the Rand health insurance study [28,29], a randomized controlled trial which found health insurance coverage, with a few exceptions, did not improve the health of adults. However, as Levy and Meltzer [2] have noted, it did not include people who previously had no health insurance; nor did it include elderly persons so the results are not comparable.

The analysis by major causes of amenable death yields results that are consistent with those found elsewhere [19]. A more detailed analysis (data not shown) revealed that the decline in circulatory diseases was largely due to a reduction in cerebrovascular and hypertensive mortality, which can plausibly be linked to a documented significant improvement in hypertension control [28] as well as, potentially, better quality of care in cerebrovascular disease. The decline in female cancer mortality can be attributed to a reduction in deaths from cervical cancer, associated with increased use of Pap smear screening [30,31], and improved outcomes with leukemia. Our finding of a significant reduction in male infectious disease mortality (-10.03% during 1997-2005) was due to a substantial decline in mortality from tuberculosis and septicemia in Taiwan during the study period (data not shown).

It is necessary to discuss what may seem like an inconsistency, whereby the causes of death making the greatest contribution to the overall decline in amenable mortality are rare in young people, who exhibit the greatest falls in amenable deaths. This is because the underlying death rate is low at young age groups, so reductions in amenable mortality from causes dominating here (that are less common at older ages) will have little impact on total amenable mortality. In contrast, a smaller reduction at older ages, where overall death rates are much higher, will allow those causes of death common in this age group to dominate amenable mortality overall.

Our study design, a large-scale (23 million population) national natural experiment using a joinpoint method, while observational in nature, allows us to compare changes in amenable mortality across different periods in time with mortality not considered amenable to health care and among different age groups with different health insurance coverage. However, the design, does not allow us to rule out all alternative explanations, and prevents us drawing causal inference, a problem identified by Levy and Meltzer in all observational studies [2]. Besides, we were not able, in this analysis, to assess the health impact of expansion of insurance coverage at the individual level. The aggregate change, which includes the 60% who already had coverage before the introduction of NHI, will therefore underestimate the impact on the previously uninsured. An earlier study did, however, find that overall life expectancy, with all its limitations as a measure of health system performance, improved more in the poorest in Taiwanese society after introduction on NHI [13].

It is difficult to explain the levelling out in the decline in amenable mortality in 1993 to 1996. Some tentative explanations can, however, be advanced. First, the rate of expansion of insurance coverage had been slowing between 1987 and 1994. Second, it took some time for the NHI to become established. It was launched in March 1995, three months into the year and unemployed people could delay enrollment for the first year. Third, it took some time for the health care delivery system to respond to the new financing model; for example, adult preventive services were not launched until April 2006. Finally, the NHI paid little attention to access by people living in remote areas until 1996. However, it may simply be that the major organizational changes involved in implementing NHI caused those working in the health care delivery system to be distracted from their core activities. This would be consistent with research on other forms of organizational change, such as hospital mergers, where the process of reorganization can lead to a loss of managerial focus and reduction in quality of care that can last for several years [32,33]. This is, however, a finding that requires further exploration.

Looking ahead, while the Taiwanese NHI has succeeded in terms of cost (3.4% of GDP), satisfaction (77.5% satisfied in 2007), low administrative cost (1.49%), and equitable financial burden [8,9], the system is not without problems. For example, as a publicly-managed program, it is difficult to insulate it from political interference, a factor that has contributed to a continuing financial deficit. Thus, the existing budget may be inadequate to sustain the current level of performance [34].

### Limitations

Our study also suffers from some limitations. First, reductions in amenable mortality may reflect a combination of three factors. These are improved access to care (for example through expansion of coverage), improved quality, and therapeutic innovation. As we have argued, it is plausible, given the timings of the changes observed, that the first of these has been important but we cannot exclude a contribution from the second and third. The only relevant data we have been able to find relate to case fatality from myocardial infarction, which improved steadily from 1996 onwards [35], but we are unable to ascertain whether this was a deviation of earlier trends. Second, as with most research on the contribution of health care to population health, by omitting potentially important factors that might reduce the underlying incidence of treatable diseases and thus the burden on the health care system. These include improvements in education, nutrition, and exposure to risk factors such as tobacco and alcohol. We were also unable to take account of possible changes in the delivery of health care or the ability to pay for care outside the NHI scheme.

Third, there are likely to be variable lag periods between expansion of coverage and reduction in amenable mortality. For example, the immediate treatment of a myocardial infarction (e.g. thrombolysis) may have an impact at once while treatment with statins or anti-hypertensives will reduce risk after a longer (and variable) period. The consequences will be to dilute any effect of the introduction of NHI.

Thus, our results must be interpreted with some caution.

### Policy Implications

These findings have implications for other countries that do not have universal health insurance coverage. The implementation of NHI in Taiwan was associated with a sustained reduction in deaths from causes amenable to health care, which surpassed the underlying decline in other causes. It is reasonable to expect that the introduction of universal coverage elsewhere might also have beneficial effects [19].

## Conclusions

Our study found that the expansion of health insurance coverage by Taiwan's NHI may have achieved one of its goals, to improve the health of the nation's population. The effect was most apparent among the age groups who were previously least likely to be insured. The major contribution to declining amenable mortality was the reduction in deaths from circulatory diseases, male infectious diseases and female treatable cancers. Our study takes advantage of a large scale natural experiment and uses the joinpoint method, to evaluate the impact of introducing NHI on population health. There are, however, some caveats. The design is not strong enough to rule out all alternative explanations than NHI. Besides, the observed gains are likely to be greater among those previously uninsured and we may have underestimated the scale of improvement because of coincident changes to recording of infant deaths.

## Abbreviations

NHI: National Health Insurance; NHE: National Health Expenditure; GDP: gross domestic product; OECD: Organisation for Economic Co-operation and Development; HPRS: Household Population Registration System; ICD9: International Classification of Diseases and Deaths; IHD: ischemic heart disease; ASMR: age-standardized mortality rates; APC: annual percentage change; FHI: Farmers Health Insurance

## Competing interests

The authors declare that they have no competing interests.

## Authors' contributions

YCL and KNK conceived the study and participated in study design, analysis and interpretation of data, wrote the article, and study supervision. YTH participated in the design of the study, analysis and interpretation of data, statistical analysis, and drafting the manuscript. SMH contributed to acquisition of data and analysis and interpretation of data. YWT, MM and EN contributed analysis and interpretation of the data and critical revision of the manuscript.

All authors read and approved the final manuscript.

## Pre-publication history

The pre-publication history for this paper can be accessed here:

http://www.biomedcentral.com/1472-6963/10/225/prepub

## References

[B1] Cheng SH Chiang TL Disparity of medical care utilization among different health insurance schemes in Taiwan Soc Sci Med 1998 47 613 620 10.1016/S0277-9536(98)00103-8 9690844

[B2] Levy H Meltzer D The impact of health insurance on health Annu Rev Public Health 2008 29 399 409 10.1146/annurev.publhealth.28.021406.144042 18031224

[B3] McWilliams JM Health consequences of uninsurance among adults in the United States: recent evidence and implications Milbank Q 2009 87 443 494 10.1111/j.1468-0009.2009.00564.x 19523125 PMC2881446

[B4] Peabody JW Yu JC Wang YR Bickel SR Health system reform in the Republic of China: formulating policy in a market-based health system JAMA 1995 273 777 781 10.1001/jama.273.10.777 7861571

[B5] Chiang TL Taiwan's 1995 health care reform Health Policy 1997 39 225 239 10.1016/S0168-8510(96)00877-9 10165463

[B6] Taiwan Department of Health Health and National Health Insurance Annual Statistics Information Service http://www.doh.gov.tw/statistic/index.htm Accessed June 1, 2009

[B7] Organisation for Economic Co-operation and Development OECD Health Data 2007 http://stats.oecd.org/Index.aspx?DataSetCode=HEALTH Accessed February 4, 2009

[B8] Cheng TM Taiwan's new national health insurance program: genesis and experience so far Health Aff (Millwood) 2003 22 3 61 76 10.1377/hlthaff.22.3.61 12757273

[B9] Lu JF Hsiao WC Does universal health insurance make health care unaffordable? lessons from Taiwan Health Aff (Millwood) 2003 22 3 77 88 10.1377/hlthaff.22.3.77 12757274

[B10] Lee YC Yang MC Li CC Health care financing system in Taiwan: before and after introduction of case-mix Malaysian J Public Health 2005 5 Suppl 2 19 32

[B11] Huang N Yip W Chang HJ Chou YJ Trends in rural and urban differentials in incidence rates for ruptured appendicitis under the National Health Insurance in Taiwan Public Health 2006 120 1055 1063 10.1016/j.puhe.2006.06.011 17011602

[B12] Chen L Yip W Chang MC Lin HS Lee SD Chiu YL Lin YH The effects of Taiwan's National Health Insurance on access and health status of the elderly Health Econ 2007 16 223 242 10.1002/hec.1160 16929478

[B13] Wen CP Tsai SP Chung WS A 10-year experience with universal health insurance in Taiwan: measuring changes in health and health disparity Ann Intern Med 2008 148 258 267 18283203 10.7326/0003-4819-148-4-200802190-00004

[B14] Davis K Huang AT Learning from Taiwan: experience with universal health insurance Ann Intern Med 2008 148 313 314 18283208 10.7326/0003-4819-148-4-200802190-00011

[B15] Nolte E McKee M Does health care save lives? Avoidable mortality revisited 2004 London: Nuffield Trust

[B16] Rutstein DD Berenberg W Chalmers TC Child CG Fishman AP Perrin EB Measuring the quality of medical care: a clinical method N Engl J Med 1976 294 582 588 10.1056/NEJM197603112941104 942758

[B17] Nolte E McKee M Measuring the health of nations: analysis of mortality amenable to health care BMJ 2003 327 1129 1132 10.1136/bmj.327.7424.1129 14615335 PMC261807

[B18] Charlton JR Hartley RM Silver R Holland WW Geographical variation in mortality from conditions amenable to medical intervention in England and Wales Lancet 1983 1 8326 Pt 1 691 696 10.1016/S0140-6736(83)91981-5 6132049

[B19] Nolte E McKee CM Measuring the health of nations: updating an earlier analysis Health Aff (Millwood) 2008 27 1 58 71 10.1377/hlthaff.27.1.58 18180480

[B20] Kim HJ Fay MP Feuer EJ Midthune DN Permutation tests for joinpoint regression with applications to cancer rates Stat Med 2000 19 335 351 10.1002/(SICI)1097-0258(20000215)19:3<335::AID-SIM336>3.0.CO;2-Z 10649300

[B21] Capewell S Morrison CE McMurray JJ Contribution of modern cardiovascular treatment and risk factor changes to the decline in coronary heart disease mortality in Scotland between 1975 and 1994 Heart 1999 81 380 386 10092564 10.1136/hrt.81.4.380 PMC1729021

[B22] Ford E Ajani U Croft J Critchley J Labarthe D Kottke T Giles W Capewell S Explaining the decrease in U.S. deaths from coronary disease, 1980-2000 N Engl J Med 2007 356 2388 2398 10.1056/NEJMsa053935 17554120

[B23] Bethesda MD Joinpoint Regression Program Version 3.2. Statistical Research and Applications Branch, National Cancer Institute. [program] Statistical Research and Applications Branch, NCI

[B24] Currie J Gruber J Saving babies: the efficacy and cost of recent changes in the Medicaid eligibility of pregnant women J Polit Econ 1996 104 1263 1296 10.1086/262059

[B25] Fox MH Moore J Davis R Heintzelman R Changes in reported health status and unmet need for children enrolling in the Kansas children's health insurance program Am J Public Health 2003 93 579 582 10.2105/AJPH.93.4.579 12660200 PMC1447793

[B26] Hadley J Waidmann T Health insurance and health at age 65: implications for medical care spending on new Medicare beneficiaries Health Serv Res 2006 41 429 451 10.1111/j.1475-6773.2005.00491.x 16584457 PMC1702516

[B27] Raghavan R Aarons GA Roesch SC Leslie LK Longitudinal patterns of health insurance coverage among a national sample of children in the child welfare system Am J Public Health 2008 98 478 484 10.2105/AJPH.2007.117408 18235059 PMC2253578

[B28] Brook RH Ware JE Rogers WH Keeler EB Davies AR Donald CA Goldberg GA Lohr KN Masthay PC Newhouse JP Does free care improve adults' health? results from a randomized controlled trial N Engl J Med 1983 309 1426 1434 10.1056/NEJM198312083092305 6355851

[B29] Keeler EB Brook RH Goldberg GA Kamberg CJ Newhouse JP How free care reduced hypertension in the health insurance experiment JAMA 1985 254 1926 1931 10.1001/jama.254.14.1926 4046121

[B30] Chen CJ You SL Lin LH Hsu WL Yang YW Cancer epidemiology and control in Taiwan: a brief review Jpn J Clin Oncol 2002 32 Suppl 1 S66 S81 10.1093/jjco/hye138 11959880

[B31] Koong SL Yen AMF Chen THH Efficacy and cost-effectiveness of nationwide cervical cancer screening in Taiwan J Med Screen 2006 13 Suppl 1 S44 S47 17227642

[B32] Fulop N Protopsaltis G King A Allen P Hutchings A Normand C Changing organisations: a study of the context and processes of mergers of health care providers in England Soc Sci Med 2005 60 119 130 10.1016/j.socscimed.2004.04.017 15482872

[B33] Sari N Do competition and managed care improve quality? Health Econ 2002 11 571 584 10.1002/hec.726 12369060

[B34] Reinhardt UE Humbled in Taiwan BMJ 2008 336 72 10.1136/bmj.39450.473380.0F 18187722 PMC2190287

[B35] Hsiao WC Chou YJ Yip WC Lu JF Li YC Evaluation of Taiwan's National Health Insurance Research report funded by the Bureau of National Health Insurance (DOH-90-NH-017), The Department of Health, Taipei, Taiwan 2003

